# Pathogen distribution, antimicrobial resistance and attributable cost analysis of neonatal sepsis in neonatal intensive care units: a propensity score matching study

**DOI:** 10.3389/fped.2025.1700766

**Published:** 2025-11-19

**Authors:** Bingyan Zhang, Peiyun Zhou, Zongke Long, Zhiwei Wang, Lan Gao, Siya Meng, Fang Xue, Xiaorong Luan

**Affiliations:** 1 School of Nursing and Rehabilitation, Cheeloo College of Medicine, Shandong University, Jinan, Shandong, China; 2 University of Health and Rehabilitation Sciences, Qingdao, Shandong, China; 3 Department of Infection Control, Qilu Hospital of Shandong University, Jinan, Shandong, China

**Keywords:** neonatal sepsis, economic burden, hospital costs, length of stay, propensity score matching

## Abstract

**Background:**

Neonatal sepsis (NS) is a serious infection in neonatal intensive care units (NICUs) that treatment challenges due to evolving antimicrobial resistance and a substantial healthcare burden. The aim of this study was to analyze the pathogenic characteristics of NS in Chinese NICUs and its independent impact on length of stay (LOS) and hospitalization costs.

**Methods:**

A retrospective case-control study was conducted including 978 neonates from two tertiary NICUs between July 1, 2023, and June 30, 2024. Propensity score matching (PSM) was used to balance the baseline characteristics between the NS and non-NS groups. Generalized linear models (GLM) were used to quantify the LOS and hospitalization costs attributable to NS. Pathogen distribution and antimicrobial resistance patterns were also assessed.

**Results:**

The incidence of NS was 8.28%. The predominant pathogens of NS were Gram-positive bacteria (71.7%), with *Staphylococcus epidermidis* (50.5%) being the predominant pathogen. Notably, multidrug-resistant (MDR) strains accounted for 65.7% of all isolates. Antimicrobial resistance analysis revealed high resistance rates of Gram-positive bacteria to penicillin G (94.6%) and oxacillin (89.3%). Gram-negative pathogens exhibited high resistance to levofloxacin (75.0%), ceftriaxone (66.7%), cefepime (66.7%), and meropenem (58.3%). After PSM, the attributable LOS for NS was 11 days (*P* = 0.002) and the attributable cost for NS was $6,035.34 (*P* < 0.001). GLM analysis showed that the LOS attributable to NS was 3.99 times longer (95% CI: 3.46–4.68) and total hospitalization costs were 1.68 times higher (95% CI: 1.42–2.00) than in non-NS patients.

**Conclusions:**

NS significantly increases the hospitalization resource consumption in NICUs. This study provides key evidence for optimizing antibiotic use strategies and advancing precision healthcare payment reform, and calls for integrating resistance surveillance with cost-control measures to reduce the health economic impact of NS.

## 1 Introduction

Neonatal sepsis (NS), a common and often fatal infection in neonatal intensive care units (NICUs), has become a major global public health challenge due to its high incidence and mortality. Epidemiological data estimate the global incidence of NS at 2,824 cases per 100,000 live births, with approximately 11%–19% of neonatal deaths attributed to NS annually (1, 2). Studies from China report that the incidence of NS in NICUs ranges from 1.8% to 15.2%, which significantly increases the risk of infant mortality and adverse outcomes (3, 4).

In recent years, advances in perinatal medicine and critical care technologies have led to improved survival of preterm infants and increased use of various invasive procedures. Consequently, the spectrum of pathogens causing NS has evolved significantly. In China, Gram-positive bacteria, particularly coagulase-negative staphylococci (CoNS) and *Staphylococcus aureus*, have emerged as the predominant pathogens (5, 6). In addition, the detection rates of multidrug-resistant (MDR) bacteria, such as methicillin-resistant *Staphylococcus epidermidis* (MRSE), have been rising annually, further complicating treatment and increasing healthcare costs (7). Therefore, clarifying the pathogen distribution and antimicrobial resistance characteristics of NS is crucial for developing effective infection control strategies, optimizing antibiotic use policies, and reducing the economic burden on healthcare systems.

Neonatal sepsis is clinically categorized based on the time of onset into early-onset (EONS, within 72 h of birth) and late-onset (LONS, after 72 h) neonatal sepsis, which have distinct pathogenesis and etiologies. EONS is primarily caused by vertical transmission of bacteria from mother to infant during delivery, with pathogens such as Group B *Streptococcus* and *Escherichia coli* being historically predominant (8–10). Its incidence shows significant geographical variation, being considerably higher in South Asia (approximately 9.8 per 1,000 live births) compared to North America (0.2–1.0 per 1,000 live births) (11–13). In contrast, LONS is mainly acquired from the postnatal hospital environment, often involving nosocomial pathogens like *Klebsiella* spp., *Staphylococcus aureus*, and fungi (14, 15). LONS is particularly prevalent among preterm and low-birth-weight infants, with up to 15% of this vulnerable population in high-income countries being affected (13). Both forms of sepsis are associated with substantial mortality, ranging from 18% to 36%, and an increased risk of adverse long-term neurodevelopmental outcomes among survivors (16–18). This distinction is critical for guiding empirical antibiotic therapy and infection control strategies.

NS not only threatens individual health but also imposes a significant economic burden on healthcare systems through resource consumption. Once present, NS can exacerbate the condition of neonates and prolong the length of stay (LOS), which reduces bed utilization efficiency and generates additional economic burdens on healthcare systems (19). However, the economic burden of sepsis has been extensively studied in adult populations (20, 21), while evidence on its health economics in neonatal populations remains scarce, particularly from China. Data from Brazil indicated that the average hospitalization cost for NS ranges from $2,970.60 to $4,305.03 (22). However, these findings may not be generalizable to different healthcare systems due to differences in national or regional economic levels, hospital levels, and medical insurance reimbursement policies. In addition, most previous studies failed to control for confounding factors such as sex, gestational age, and birth weight, which may introduce bias into cost assessments. Therefore, this study aims to establish comparable groups of septic and non-septic neonates using propensity score matching (PSM) to quantify the impact of NS on NICU LOS and hospitalization costs. Meanwhile, by integrating data on pathogenetic characteristics, antimicrobial resistance patterns and economic burden results of NS, the findings collectively provide localized evidence to optimize infection control strategies and rationalize healthcare resource allocation in the NICU.

## 2 Materials and methods

### 2.1 Study design and population

This retrospective case-control study was conducted in the NICUs of two university-affiliated tertiary hospitals in Shandong Province, China, between July 1, 2023, and June 30, 2024. The obstetric ward of each hospital admits approximately 5,000–7,000 births per year, and the NICU admits over 1,500 neonates annually. The case group included neonates diagnosed with NS, and the control group comprised non-NS neonates hospitalized during the same period. To compare the differences in LOS and hospitalization costs between the two groups, confounders were controlled through PSM. All neonates admitted to the NICU aged ≤28 days during the study period were included in this study. Exclusion criteria were as follows: (1) neonates with severe congenital malformations or inherited metabolic diseases; (2) neonates with concurrent severe infections in other organs, as these conditions can independently prolong hospital stays and increase costs, thereby confounding the attribution of resource use solely to sepsis; (3) neonates who died or were discharged within 24 h of admission, to ensure a sufficient observation period for assessing the development and impact of NS; and (4) neonates with incomplete medical records. The study was approved by the Ethics Committee of Shandong University (2024-R-174). As a non-interventional observational study, the committee waived the requirement for informed consent.

### 2.2 Diagnostic criteria for NS

The diagnosis of NS was based on the Expert Consensus on Diagnosis and Management of Neonatal Bacteria Sepsis (2024) (23). Confirmed cases required pathogen identification through blood culture or sterile cavity fluid culture (e.g., cerebrospinal fluid), with clinical manifestations consistent with the identified pathogens. According to the time of onset, NS was classified into early-onset sepsis (EONS, occurring ≤72 h after birth) and late-onset sepsis (LONS, occurring >72 h after birth).

### 2.3 Data collection

Demographic data (sex, gestational age, birth weight, Apgar scores at 1 and 5 min, mode of delivery) and perinatal risk factors (duration of rupture of membranes, meconium-stained amniotic fluid) were collected from the hospital electronic medical record system for all participants. Outcome variables included total LOS and hospitalization costs. The total LOS was defined as the difference between the date of admission and discharge (in days). The total hospitalization costs covered direct medical expenses, including comprehensive medical service category (general medical service costs, general treatment operating costs, nursing costs), diagnosis category (pathological diagnosis costs, laboratory diagnostic costs, imaging diagnostic costs, clinical diagnosis project costs), treatment category (non-surgical clinical physiotherapy costs, surgical treatment costs), Traditional Chinese Medicine (TCM) costs (refers to costs incurred from treatments using TCM techniques or herbal medicines), medication category (western medicine costs, which includes antibacterial drug costs), blood product costs, consumable material category (treatment disposable medical materials costs and Surgical disposable medical materials costs). The totals were converted to US dollars based on the 2024 exchange rate (1 US$ = 7.1217 RMB). In addition, NS-related pathogenic data, including infection onset time, pathogen type, antimicrobial susceptibility testing results and MDR status [defined as bacteria resistant to at least one agent in three or more antimicrobial categories (24)], were extracted from the hospital infection management information system.

### 2.4 Statistical analysis

A logistic regression model was used to calculate the propensity score, with NS as the dependent variable and covariates including sex, gestational age, birth weight, Apgar scores, mode of delivery, duration of rupture of membranes, and meconium-stained amniotic fluid. The matching process was performed using the “MatchIt” R package with optimal matching at a 1:2 ratio between cases and controls to ensure balanced baseline characteristics. Continuous variables were described as mean ± standard deviation or median (interquartile range, IQR) based on data distribution, while categorical variables were expressed as frequency (percentage). Differences between continuous variables were compared using the two independent samples *t*-test or Mann–Whitney *U* test, and differences between categorical variables were compared using the chi-square or Fisher's exact tests.

Then, generalized linear models (GLMs) were constructed with LOS (negative binomial distribution) or hospitalization costs (gamma distribution) as the dependent variable, using a log-link function. All covariates and NS occurrence were included as independent variables. The missing data were imputed using the multiple imputation approach implemented with the “mice” package in R. Data were analyzed using R software 4.4.1 (R Foundation for Statistical Computing). For standard analyses, a two-sided *P* < 0.05 was considered to indicate statistical significance.

## 3 Results

### 3.1 Patient inclusion

**Figure 1:**
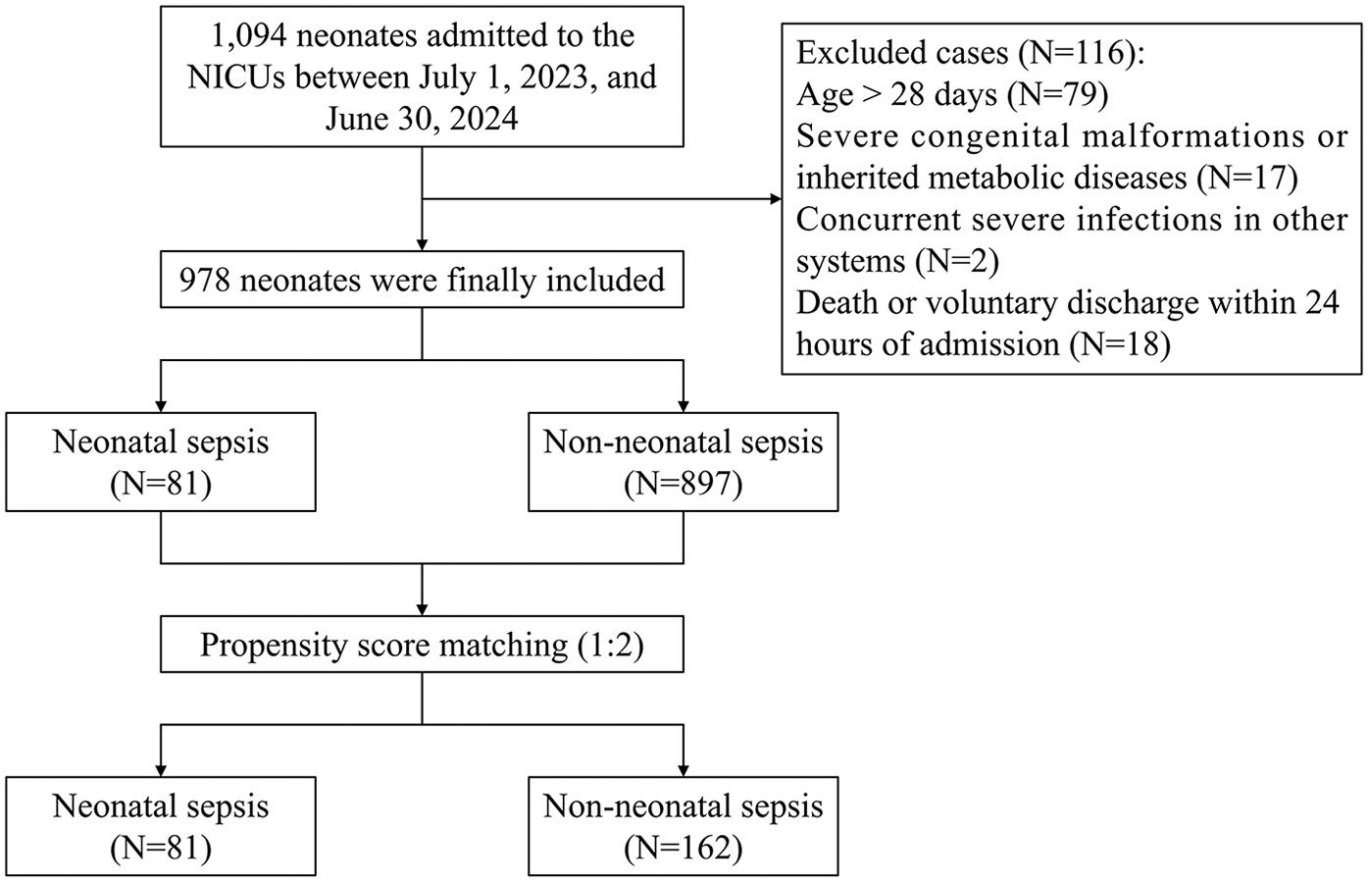
Flow chart of patient selection and study design.

A total of 1,094 neonates were admitted to the NICUs of the two hospitals between July 1, 2023, and June 30, 2024. After excluding 116 neonates who did not meet the inclusion criteria, 978 neonates were finally included. Throughout the study period, only three in-hospital deaths (0.3%) were recorded. However, a notably high rate of discharge against medical advice was observed, accounting for 4.6% (45/978) of the cohort. Before matching, the cohort comprised 81 neonates in the NS group and 897 in the non-NS group. Following 1:2 PSM, there were 81 in the NS group and 162 in the non-NS group. Figure 1 presents a flow chart of patient selection and study design.

### 3.2 Pathogen distribution of neonatal sepsis

Among the 978 neonates included, 81 were diagnosed with NS, with an incidence of 8.28%. A total of 99 pathogens were isolated. Gram-positive bacteria (71.7%) were predominant, with *S. epidermidis* (50.5%) being the most common. As detailed in Table 1, the pathogen profile differed between EONS and LONS. Although Gram-positive bacteria were predominant in both groups, they constituted a higher proportion in LONS (83.3%) than in EONS (69.1%). Conversely, Gram-negative bacteria were more prevalent in EONS (28.4%) than in LONS (16.7%).

**Table 1:** Distribution of pathogens in early- and late-onset neonatal sepsis.

Isolated pathogens	Total *N* (%)	EONS *N* (%)	LONS *N* (%)
Gram-positive	71 (71.7)	56 (69.1)	15 (83.3)
*Staphylococcus epidermidis*	50 (50.5)	43 (53.1)	7 (38.9)
*Staphylococcus aureus*	3 (3.0)	0 (0.0)	3 (16.7)
*Staphylococcus hominis*	3 (3.0)	3 (3.7)	0 (0.0)
*Bacillus cereus*	3 (3.0)	3 (3.7)	0 (0.0)
*Enterococcus faecium*	3 (3.0)	1 (1.2)	2 (11.1)
*Staphylococcus warneri*	3 (3.0)	2 (2.5)	1 (5.6)
*Staphylococcus haemolyticus*	2 (2.0)	1 (1.2)	1 (5.6)
*Staphylococcus capitis*	1 (1.0)	1 (1.2)	0 (0.0)
*Streptococcus parasanguinis*	1 (1.0)	1 (1.2)	0 (0.0)
*Streptococcus agalactiae*	1 (1.0)	0 (0.0)	1 (5.6)
*Listeria monocytogenes*	1 (1.0)	1 (1.2)	0 (0.0)
Gram-negative	26 (26.3)	23 (28.4)	3 (16.7)
*Klebsiella pneumoniae*	5 (5.1)	5 (6.2)	0 (0.0)
*Pseudomonas aeruginosa*	5 (5.1)	5 (6.2)	0 (0.0)
*Escherichia coli*	4 (4.0)	3 (3.7)	1 (5.6)
*Acinetobacter baumannii*	3 (3.0)	3 (3.7)	0 (0.0)
*Acinetobacter nosocomialis*	3 (3.0)	3 (3.7)	0 (0.0)
*Ralstonia mannitolilytica*	2 (2.0)	1 (1.2)	1 (5.6)
*Klebsiella oxytoca*	1 (1.0)	0 (0.0)	1 (5.6)
*Citrobacter freundii*	1 (1.0)	1 (1.2)	0 (0.0)
*Stenotrophomonas maltophilia*	1 (1.0)	1 (1.2)	0 (0.0)
*Pantoea agglomerans*	1 (1.0)	1 (1.2)	0 (0.0)
Fungi	2 (2.0)	2 (2.5)	0 (0.0)
*Candida parapsilosis*	1 (1.0)	1 (1.2)	0 (0.0)
*Aspergillus flavus*	1 (1.0)	1 (1.2)	0 (0.0)
Total	99 (100.0)	81 (100.0)	18 (100.0)

EONS, early-onset neonatal sepsis; LONS, late-onset neonatal sepsis.

Further stratification by gestational age revealed that the incidence of NS was significantly higher in preterm infants (69/586, 11.77%) than in term infants (12/392, 3.06%). The etiology also varied by gestational age group. In preterm infants, the vast majority of cases were EONS (64/69, 92.8%), characterized by a high prevalence of Gram-positive bacteria (68.8% of isolates). In contrast, term infants with NS were more likely to develop LONS (8/12, 66.7%), which also exhibited a Gram-positive dominant profile (84.6% of isolates).

In addition, most of the isolated pathogens were found to be MDR, with 65 of 99 isolates classified as MDR pathogens. The most frequent MDR bacteria included methicillin-resistant *S. epidermidis* (43/65), carbapenem-resistant *K. pneumoniae* (4/65), extended-spectrum β-lactamase-producing *Escherichia coli* (4/65), and carbapenem-resistant *P. aeruginosa* (4/65).

### 3.3 Antimicrobial resistance of neonatal sepsis pathogens

**Figure 2:**
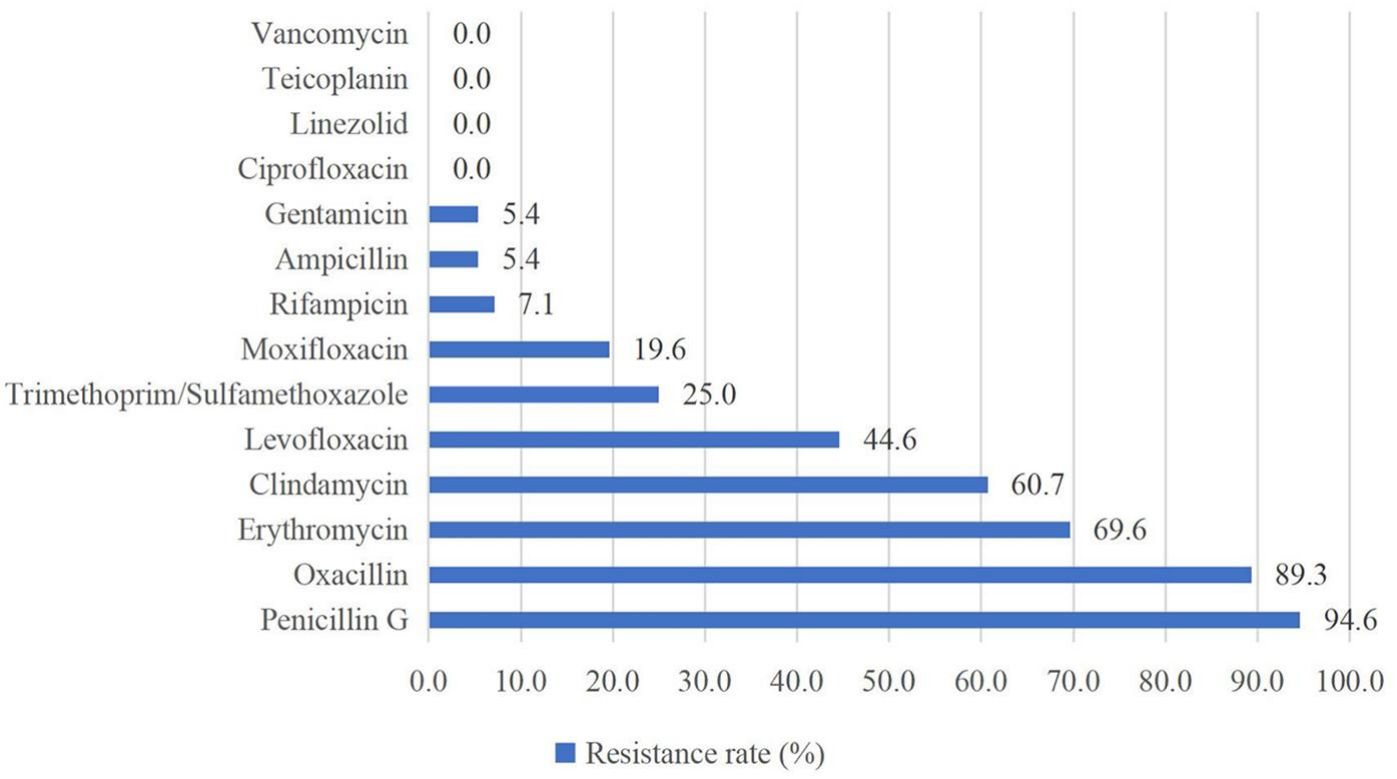
Antimicrobial resistance pattern of isolated gram-positive bacteria.

**Figure 3:**
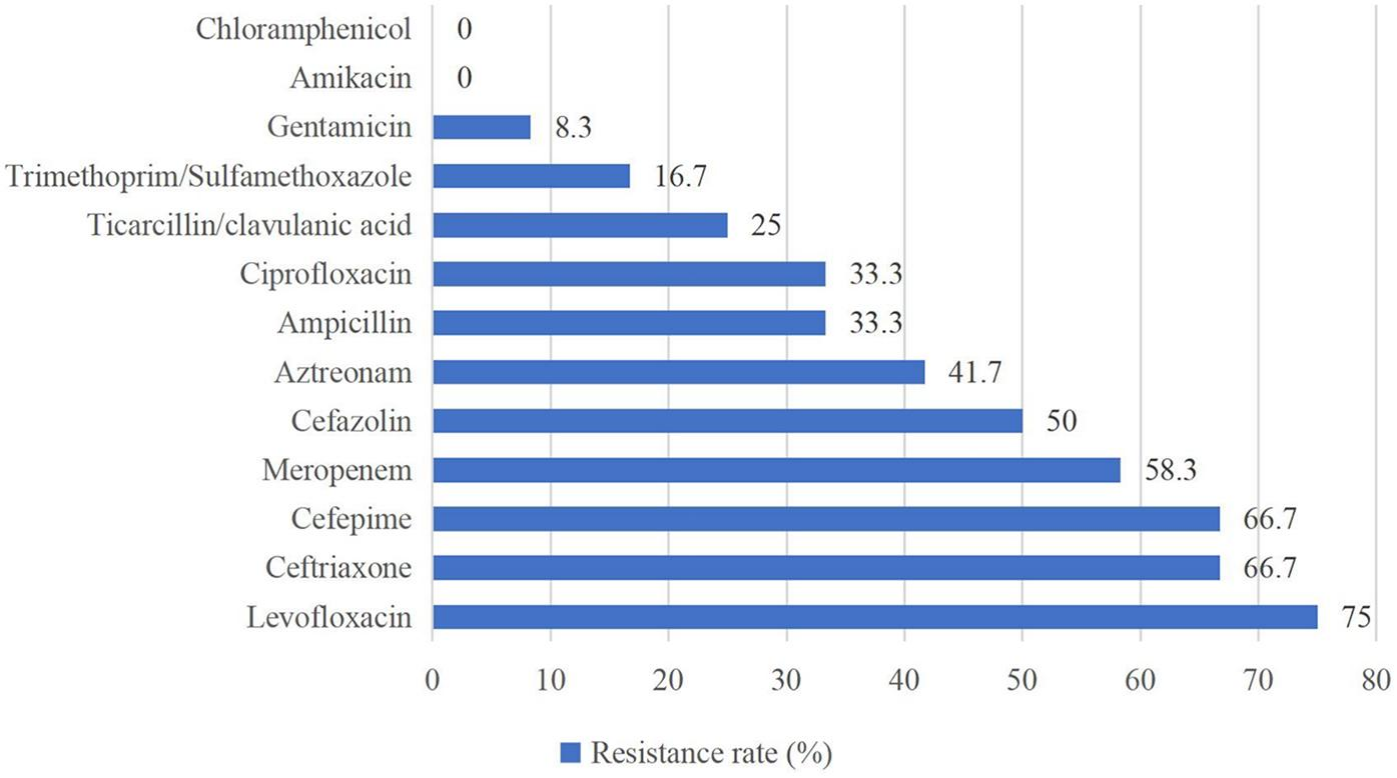
Antimicrobial resistance pattern of isolated gram-negative bacteria.

Of the Gram-positive bacteria detected, almost all strains were resistant to penicillin G (94.6%) and oxacillin (89.3%). None of the strains were resistant to vancomycin, teicoplanin, linezolid, or ciprofloxacin (Figure 2). For Gram-negative bacteria, high resistance rates (above 50%) were observed for levofloxacin (75.0%), ceftriaxone (66.7%), cefepime (66.7%), and meropenem (58.3%). Gram-negative bacteria showed the highest susceptibility to chloramphenicol and amikacin (Figure 3).

### 3.4 Baseline characteristics before and after PSM

The baseline characteristics of the study participants are summarized in Table 2. Among all included neonates, males were more common, and most deliveries were by cesarean section. Before matching, the NS group and the non-NS group differed significantly in several baseline clinical characteristics. The NS group had a higher proportion of extremely preterm infants (<28 weeks: 25.9% vs. 2.9%) and extremely low birth weight infants (≤1,000 g: 29.6% vs. 4.0%), lower Apgar scores at 1 min (<7: 34.6% vs. 8.2%), and higher rates of meconium-stained amniotic fluid (23.5% vs. 11.6%) (all *P* < 0.05). After 1:2 PSM, all variables were balanced between the case and control groups with no significant differences (all *P* > 0.05).

**Table 2:** Baseline characteristics before and after matching.

Characteristic	Before matching			After matching		
	NS (*N* = 81)	Non–NS (*N* = 897)	*P*	NS (*N* = 81)	Non–NS (*N* = 162)	*P*
Sex			0.776			0.964
Male	44 (54.3)	508 (56.6)		44 (54.3)	90 (55.6)	
Female	37 (45.7)	389 (43.4)		37 (45.7)	72 (44.4)	
Gestational age (weeks)			<0.001			0.328
<28	21 (25.9)	26 (2.9)		21 (25.9)	26 (16.0)	
28–31^+6^	30 (37.0)	105 (11.7)		30 (37.0)	69 (42.6)	
32–36^+6^	18 (22.2)	386 (43.0)		18 (22.2)	42 (25.9)	
≥37	12 (14.8)	380 (42.4)		12 (14.8)	25 (15.4)	
Birth weight (g)			<0.001			0.496
≤1,000	24 (29.6)	36 (4.0)		24 (29.6)	35 (21.6)	
1,001–1,500	30 (37.0)	84 (9.4)		30 (37.0)	74 (45.7)	
1,501–2,500	12 (14.8)	312 (34.8)		12 (14.8)	23 (14.2)	
>2500g	15 (18.5)	465 (51.8)		15 (18.5)	30 (18.5)	
Apgar at 1 min			<0.001			0.174
<7	28 (34.6)	74 (8.2)		28 (34.6)	41 (25.3)	
≥7	53 (65.4)	823 (91.8)		53 (65.4)	121 (74.7)	
Apgar at 5 min			0.080			1.000
<7	3 (3.7)	8 (0.9)		3 (3.7)	5 (3.1)	
≥7	78 (96.3)	889 (99.1)		78 (96.3)	157 (96.9)	
Mode of delivery			0.715			0.641
Vaginal delivery	23 (28.4)	232 (25.9)		23 (28.4)	40 (24.7)	
Cesarean delivery	58 (71.6)	665 (74.1)		58 (71.6)	122 (75.3)	
Duration of rupture of membranes (h)			0.719			0.709
<18	74 (91.4)	802 (89.4)		74 (91.4)	144 (88.9)	
≥18	7 (8.6)	95 (10.6)		7 (8.6)	18 (11.1)	
Meconium-stained amniotic fluid			0.004			0.220
No	62 (76.5)	793 (88.4)		62 (76.5)	136 (84.0)	
Yes	19 (23.5)	104 (11.6)		19 (23.5)	26 (16.0)	

NS, neonatal sepsis.

### 3.5 Comparison of LOS and hospitalization costs after PSM

After PSM, the median LOS in the case group was 45 days, significantly longer than the 34 days in the control group (*P* = 0.002), with a median difference of 11 days. The median total hospitalization costs were $15,003.24 and $8,967.90 for the case and control groups, respectively, with a difference of $6,035.34 (*P* < 0.001). Comparison of hospitalization costs between groups showed statistically significant differences in all categories except for non-surgical clinical physiotherapy costs and TCM costs, as detailed in Table 3.

**Table 3:** Comparison of length of stay (days) and hospitalization cost (US$) between the matched pairs (neonatal sepsis group vs. control group).

Variable	Cases, median (IQR)	Controls, median (IQR)	Statistics Z	*P*
Length of stay	45 (27–66)	34 (13–53)	−3.027	0.002
Total hospitalization costs	15,003.24 (7,677.09–21,895.28)	8,967.90 (3,115.47–14,538.27)	−4.206	<0.001
General medical service costs	2,164.37 (1,212.07–3,345.83)	1,663.09 (653.50–2,662.92)	−2.946	0.003
General treatment operating costs	2,443.29 (905.35–5,310.40)	1,241.13 (358.74–3,159.39)	−3.733	<0.001
Nursing costs	1,872.73 (882.23–3,112.74)	1,325.25 (413.81–2,368.29)	−3.020	0.003
Pathological diagnosis costs	42.12 (21.06–63.19)	0.00 (0.00–21.06)	−8.138	<0.001
Laboratory diagnostic costs	1,398.95 (864.70–2,099.29)	817.47 (449.95–1,277.07)	−5.331	<0.001
Imaging diagnostic costs	414.23 (258.37–593.54)	248.54 (143.22–449.33)	−4.144	<0.001
Clinical diagnosis project costs	155.16 (92.39–339.39)	86.43 (50.16–157.23)	−4.459	<0.001
Non-surgical clinical physiotherapy costs	975.26 (277.18–1,543.17)	595.85 (126.90–1,245.73)	−1.741	0.082
Surgical treatment costs	25.27 (10.11–87.06)	13.06 (6.74–19.66)	−3.961	<0.001
Traditional Chinese Medicine costs	0.00 (0.00–42.12)	0.00 (0.00–0.00)	−0.806	0.42
Western medicine costs	2,124.58 (963.13–3,476.51)	1,166.19 (192.19–1,948.44)	−4.676	<0.001
Antibacterial drug costs	683.66 (221.38–1,070.64)	69.47 (29.14–188.01)	−8.352	<0.001
Blood product costs	73.02 (36.51–561.66)	36.51 (0.00–72.31)	−6.042	<0.001
Treatment disposable medical materials costs	1,409.73 (699.76–2,144.54)	886.53 (226.25–1,446.79)	−4.252	<0.001
Surgical disposable medical materials costs	338.57 (149.42–500.92)	142.61 (49.44–276.93)	−5.311	<0.001
Other	170.04 (105.49–243.20)	138.84 (76.07–202.45)	−2.515	0.012

IQR, interquartile range.

### 3.6 GLM analysis of LOS and hospitalization costs between groups

In the negative binomial regression model, the LOS attributable to NS was 3.99 times (95% CI: 3.46–4.68, *P* < 0.001) higher than that in non-NS infants. Gamma regression analysis showed that total hospitalization costs attributable to NS were 1.68 times (95% CI: 1.42–2.00, *P* < 0.001) higher than those in non-NS infants, as shown in Table 4.

**Table 4:** Generalized linear model analysis of neonatal sepsis-associated length of stay (days) and total hospitalization costs (US$).

Variable	Estimate	95% CI	*P*
Length of stay	3.99	3.46–4.68	<0.001
Total hospitalization costs	1.68	1.42–2.00	<0.001

CI, confidence interval.

## 4 Discussion

This study systematically assessed the impact of NS on hospitalization resource consumption by constructing a comparable cohort through PSM. Our findings revealed an NS incidence of 8.28% among all infants admitted to the NICUs during the study period, with *S. epidermidis* as the predominant pathogen. Post-PSM analysis demonstrated that NS prolonged hospitalization by 11 days and increased total costs by $6,035.34. GLM analysis quantified the attributable effects of NS, demonstrating a 3.99-fold increase in LOS and a 68% higher total hospitalization cost compared to non-NS infants.

Globally, the incidence of NS shows significant geographical heterogeneity (0.30%–17.0%) (1, 25). In this study, the NS incidence was 8.28%, which was higher than the prevalence reported in a multicenter study covering seven low- and middle-income countries (LMICs) (4.69%) (26). This discrepancy may be attributed to differences in preterm birth rates and the proportion of low birth weight (LBW) infants in different countries, as well as to socioeconomic disparities affecting access to healthcare resources and the quality of perinatal care. Due to the specific nature of the hospitals included in this study as regional tertiary referral centers, they admitted more preterm and LBW infants, suggesting that our study population had greater clinical severity. It is important to note that our incidence estimate was limited to neonates admitted to NICUs and did not cover potential cases in community or general obstetrics wards, which may overestimate the actual burden of disease at the population level. Notably, the in-hospital mortality was low (0.3%), but a substantial number of families chose discharge against medical advice. This reflects scenarios where families may have opted to withdraw treatment due to the infant's critical condition, financial constraints, or a perceived poor prognosis. Thus, the true mortality burden may be higher than recorded.

Pathogenic analysis showed that Gram-positive bacteria (71.7%) were the major pathogens in our study, with CoNS being the most prevalent, a finding consistent with reports from other tertiary NICUs (4, 7, 27). This contrasts sharply with the Gram-negative bacterial dominance commonly reported in studies from LMICs (28–31), highlighting the influence of healthcare settings on etiology. Our findings confirm the significant epidemiological and etiological distinctions between EONS and LONS. The higher incidence of EONS in our study aligns with its established link to maternal peripartum factors and vertical transmission. Given this established link, the management of conditions like preterm premature rupture of membranes (PPROM) becomes essential. Current obstetrical guidelines universally advocate for the use of antibiotic prophylaxis in cases of PPROM, a practice aimed at reducing the incidence of EONS (32). Our data indicate that EONS imposes a significant burden, underscoring the value of this evidence-based intervention. In contrast, the lower incidence of LONS likely reflects its association with prolonged hospitalization and invasive procedures in a more selected, high-risk preterm population.

Notably, the particularly high rate of *S. epidermidis* in EONS, especially among preterm infants, highlights its role as a primary opportunistic pathogen in the NICU environment. Its ability to form biofilms on medical device surfaces and resist a wide range of disinfectants provides a transmission advantage in NICUs where invasive procedures are frequent (33). The notable absence of Group B Streptococcus in our EONS cases likely reflects the successful implementation of maternal screening and intrapartum antibiotic prophylaxis.

The absence of fungal pathogens in our LONS cases may be partly attributable to the 12-month study period. Given the declining incidence and sporadic nature of invasive fungal infections in neonates in recent years (34), a longer surveillance period is often required to adequately capture their true prevalence. Additionally, the routine use of antifungal prophylaxis in high-risk infants and strict infection control practices in our unit may have further contributed to this observed absence. Nevertheless, continuous surveillance remains imperative, as fungal sepsis continues to be a significant threat in NICUs worldwide.

The stratification by gestational age unveiled critical insights. Preterm infants constituted the overwhelming majority of our sepsis cases, primarily presenting with EONS. This underscores the paramount role of prematurity and its associated immune immaturity as the principal risk factor for NS (8, 35). Conversely, term infants with sepsis in our study predominantly developed LONS, a finding that warrants further investigation but may relate to specific nosocomial exposures or unmeasured clinical complexities. This stark contrast emphasizes that the profile of neonatal sepsis in a given population is largely dictated by the proportion of preterm births, necessitating tailored prevention strategies for different gestational age groups.

Antimicrobial susceptibility testing indicated that Gram-positive bacteria showed widespread resistance to β-lactam antibiotics (penicillin G 94.6%, oxacillin 89.3%), which is consistent with findings from other studies (36, 37). Additionally, 100% of Gram-positive bacteria in this study remained susceptible to vancomycin, teicoplanin, linezolid, and ciprofloxacin, similar to the results reported by Tessema et al. (36). These findings provide critical therapeutic options for Gram-positive bacterial infections, but these “last-resort” antibiotics need to be used with caution to avoid the development of resistance. Both our study and studies in other regions demonstrated high susceptibility of Gram-negative bacteria to chloramphenicol and amikacin (37–39), suggesting that these two drugs may be considered as potential antibiotics for the empirical treatment of NS caused by Gram-negative bacteria in the future, but their inherent toxicities (e.g., gray baby syndrome, ototoxicity) necessitate rigorous risk-benefit evaluation. Furthermore, the high percentage of MDR strains (65.7%), predominantly MRSE, may be closely related to *mecA*-mediated β-lactam resistance and selection pressure for broad-spectrum antibiotics in the hospital setting (40). In addition, MRSE enhances colonization through biofilm formation and may acquire additional resistance genes (e.g., aminoglycoside-modifying enzymes) through horizontal gene transfer, further entrenching its MDR phenotype (41).

Our study quantified the independent impact of NS on healthcare resources through PSM. The median LOS for infants with NS was 45 days, and the median total hospitalization cost was $15,003. A systematic review of sepsis studies in several countries (21) reported that the average total hospitalization cost for sepsis patients in China was $13,292, which was similar to our findings. However, another systematic review that included 20 studies from high- and middle-income countries (HMICs) showed that the cost of sepsis treatment in all middle-income countries ($55**–**$3,607) was much lower than that in the United States ($64,047**–**$129,632) (42). This disparity stems from the differences in healthcare cost allocation, treatment strategies, and medical insurance systems between countries of different income levels. High-income countries are dominated by expenditures on labor costs, advanced antibiotics, and organ support technologies, while middle-income countries face higher spending on medications and consumables due to antimicrobial resistance challenges and resource constraints. At the same time, variations in insurance coverage, reimbursement rates, and payment models in different countries further influence patient financial burdens and healthcare cost control. In addition, differences in LOS can further exacerbate cost differentiation, as the above review (42) noted that high-income countries reported an average LOS of 24–72 days, while middle-income countries reported only 4–14 days. In our study, the LOS (45 days) and total cost ($15,003) were intermediate. As a upper-middle-income country, China's healthcare system has unique characteristics. Whereas regional disparities in healthcare resources have led to the concentration of critically ill infants in tertiary hospitals similar to our study sites, prolonging LOS, medical insurance cost-containment policies such as centralized procurement and diagnosis-related groups (DRGs)/disease intervention package (DIP) payment reforms have partially reduced healthcare costs for both patients and hospitals.

Our study also showed that the NS group had significantly higher expenditures in each medical cost subcategory, except for non-surgical clinical physiotherapy costs and TCM costs. The greatest differences were observed in general treatment operating costs, western medicine costs, and laboratory diagnostic costs, consistent with the results of a related study in China (43). The high expenditure of general treatment operating costs stemmed from frequent invasive procedures and intensive care required by NS infants due to their critical health status. The disparity in western medicine costs was primarily caused by antibacterial drug costs, as NS management necessitates the long-term use of broad-spectrum antibiotics, and the prevalence of multidrug-resistant bacteria often forces clinics to use expensive combination regimens. In addition, the increase in laboratory diagnostic costs may be associated with additional pathogen identification tests, repeated blood cultures, and dynamic monitoring of inflammatory markers (e.g., procalcitonin, IL-6), which are essential for guiding antibiotic therapy but significantly increase the frequency and costs of testing. Therefore, while ensuring precision in NS diagnosis and treatment, it is still necessary to rationally control the costs of general treatment operations, antimicrobial drug use, and laboratory diagnostics to optimize resource utilization and reduce patient financial burdens.

This study has several limitations. First, as a retrospective observational study, although PSM was applied to control for known confounders, the potential influence of unmeasured confounders cannot be fully excluded. For instance, missing data on perinatal antibiotic exposure preclude a detailed analysis of its impact on initial microbial colonization and subsequent pathogen characteristics in sepsis cases, which represents a potential confounding factor. Second, the data were obtained from only two tertiary hospitals, potentially leading to overestimation of NS incidence due to the concentration of critically ill neonates. Future studies should include data from community hospitals or general obstetrics wards to fully reflect the epidemiological characteristics of NS. Third, the cost analysis did not include indirect costs (e.g., family productivity loss and long-term sequelae costs), which may have led to underestimation of the overall economic burden of NS. Finally, the 12-month study period limited the assessment of seasonal epidemiological changes or long-term antimicrobial resistance trends. Prospective multicenter cohort studies are needed in the future to comprehensively evaluate the health economic impacts of NS and the dynamics of antimicrobial resistance transmission.

## 5 Conclusion

This study systematically assessed the independent impact of NS on NICU LOS and hospitalization costs through the PSM approach and provided localized evidence of NS-attributable costs in the Chinese context. The findings demonstrated that NS had a significant impact on healthcare resource consumption, highlighting its role as a major health economic challenge in NICUs. The study also suggests that policymakers to combine antimicrobial resistance surveillance and cost-containment strategies to promote the implementation of precision health economy policies.

## Data Availability

The raw data supporting the conclusions of this article will be made available by the authors, without undue reservation.
